# Ultrasound-Mediated Microbubble Destruction Inhibits Skin Melanoma Growth by Affecting YAP1 Translation Using Ribosome Imprinting Sequencing

**DOI:** 10.3389/fonc.2021.619167

**Published:** 2021-04-29

**Authors:** Tianhong Wei, Lan Li, Zhiyou He

**Affiliations:** ^1^ Department of Ultrasonography, Xiangya Hospital, Central South University, Changsha, China; ^2^ Department of Burns and Reconstructive Surgery, Xiangya Hospital, Central South University, Changsha, China

**Keywords:** ultrasound-mediated microbubble destruction, skin melanoma, ribosomal blot sequencing, drug resistance, YAP1

## Abstract

Cutaneous melanoma (CMM) is a skin tumor with a high degree of malignancy. BRAF resistance imposes great difficulty to the treatment of CMM, and partially contributes to the poor prognosis of CMM. YAP is involved in the growth and drug resistance of a variety of tumors, and mechanical signals may affect the activation of YAP1. As a novel ultrasound treatment technology, ultrasound-mediated microbubble destruction (UMMD) has been reported to have a killing effect on isolated CMM cells. In this study, the tumor tissue samples were collected from 64 CMM patients. We found that YAP1 mRNA expression was irrelevant to the clinicopathological characteristics and prognostic survival of the CMM patients. The drug-resistant cell line was constructed and subcutaneously implanted into nude mice, which were further separately treated with UMMD, ultrasound (US), and microbubbles (MB). The result showed that UMMD significantly inhibited the growth of tumor tissues. Ribosome imprinting sequencing (Ribo-seq) is a genetic technology for studying protein translation at genetic level. Ribo-seq, RNA-seq, and RT-qPCR were applied to detect YAP1 expression in CMM mouse tumor tissues. Ribo-seq data revealed that UMMD greatly up-regulated the expression of YAP1, interestingly, the up-regulated YAP1 was found to be negatively correlated with the weight of tumor tissues, while no significant change in YAP1 expression was detected by RNA-seq or RT-qPCR assay. These results indicated that UMMD could inhibit the tumor growth of drug-resistant CMM by affecting the translation efficiency of YAP1, providing a strong basis for the clinical treatment of UMMD in CMM.

## Introduction

Cutaneous melanoma (CMM) is a skin tumor with a high degree of malignancy and rising incidence (1, 2), with a larger tumor volume positively correlating with an increased prognostic risk of patients (3). Thus, in the related field, exploring the mechanisms underlying the occurrence and development CMM have become a main research direction for improve CMM patients’ prognosis. The discovery of missense mutation of the BRAF gene is one of the most influential developments in the study of CMM, as inn more than half of CMM cases, the valine at position 600 of the BRAF protein is replaced by glutamic acid (BRAF^V600E^) (4, 5). Studies found that BRAF^V600E^ mutation is related to a higher chance of developing tumor metastasis and lower survival rate of CMM patients (5, 6). As a potent kinase inhibitor selectively targeting BRAF^V600E^ mutation in tumor cells, Verofini (PLX 4032) has a therapeutic effect on patients with metastatic melanoma with BRAF^V600E^ mutations and could improve the overall survival of CMM patients (7). However, PLX 4032 is prone to develop drug resistance within 6-8 months of treatment, greatly imposing the difficulty of clinical treatment of CMM patients (8).

Recent studies have demonstrated that the activation and promotion of the core transcription factor YAP in the hippo signaling pathway (9) enhance drug resistance in anti-cancer treatments. Under PLX 4032 treatment, drug-resistant melanoma cells shows a higher level of YAP nuclear localization and transcription activity (10). Co-treatment of inhibition of YAP1 activity and PLX 4032 has been confirmed as a feasible treatment for BRAF-resistant melanoma derived from cancer stem cells (11). Previous study also points out that the increase of YAP1 in tumors with BRAF^V600E^ mutation is a biomarker indicative of poor early response of patients (12), suggesting the potential of the expression of YAP1 in PLX 4032 drug-resistant melanoma as a novel research direction.

Ultrasound-mediated microbubble destruction (UMMD) is an effective technology with minimal invasiveness. By combining low-frequency ultrasound with microbubbles, cavitation, that is, pushing and pulling or shock waves, will be generated in the body. Cavitation is expected to provide a safe and effective new anti-cancer therapy for the clinical practice, because it can produce a biological barrier penetration, improve the efficiency of drug or gene delivery into tumor tissues, and activate the anti-tumor immune response (13, 14). It is well-known that YAP1 is the hub for integrating multiple mechanical signals, which can then affect cell fate by mediating YAP1 (15). We suspected that UMMD may affect CMM growth by changing YAP cell activity.

This experiment was the first to explore the clinical significance of YAP1 in CMM. The tumor tissue samples were collected from CMM patients, and we constructed a CMM animal model with PLX 4032 resistance to analyze the effect of UMMD treatment on YAP1 expression *in vivo*. Noticeably, in addition to conventional RNA-seq and QPCR, this study also performed ribosomal blot sequencing (Ribo-seq), which directly detects protein translation at the gene level (16) for better demonstrating the effect of UMMD on YAP1 activity.

## Materials and Methods

### Research Object

A total of 64 patients aged 16-68 years old who underwent CMM surgery at XIANGYA HOSPITAL CENTRAL SOUTH UNIVERSITY hospital between January 2014 and August 2015 were selected as research subjects. All the patients were clinically and pathologically diagnosed as having CMM. Patients’ complete case data and tissue samples were collected. Treatment and follow-up were all conducted according to the research guidelines, and patients’ survival was closely recorded. The general information of patients is shown in **Table 1**. This study has been approved by the Medical Ethics Committee of our hospital.

**Table 1:** Clinicopathological characteristics of patients.

	Number	Percentage (%)
age		
≤50	26	40.62
>50	38	59.38
sex		
male	29	45.31
femal	35	54.69
Lesion		
neck	9	14.06
trunk	17	26.56
others	38	59.38
Cell subtype		
Epithelioid	36	56.25
Fusocellular	28	43.75
Organization Type		
Nodular	16	25.00
other	48	75.00
Clark classification		
I-III	18	28.12
IV-V	46	71.88
Breslow thickness (mm)		
≤2	19	29.69
>2	45	70.31
BRAF (V600E) mutation		
no	33	51.56
yes	31	48.44
Tumor growth phase		
Radial	10	15.62
Vertical	54	84.38
Mitosis count		
≤5/HPF	42	65.62
>5/HPF	22	34.38

### RT-qPCR

RNA extraction kit (GBCBIO, R3105) was used to separate and purify RNA in the CMM tissues collected. M-MLV 4 reverse transcription kit (Biomed, MT403) was applied to synthesize first strand cDNA from RNA templates. TransScript^®^ Green Two-Step Kit (Trans, AQ201) was employed for QPCR amplification. GAPDH served as an internal reference. See **Table 2** for the specific primer sequences used in the experiment.

**Table 2:** Primer sequence.

Gene	Primer	Sequence (5'-3')	Length	Location
YAP1	Forward	TTTTACCGCGTCTCCCTGATT	21	607-627
	Reverse	AGAAACACCTGGGCTAGTAGAAA	23	819-797
GAPDH	Forward	GGAGCGAGATCCCTCCAAAAT	21	108-128
	Reverse	GGCTGTTGTCATACTTCTCATGG	23	304-282

### Construction of Drug-Resistant Cell Lines

The human CMM cell A375 was commercially purchased from the Cell Bank of the Chinese Academy of Sciences. Fenghui was entrusted to establish PLX 4032 drug-resistant A375 cells, which successfully survived as a drug-resistant strain (PLX4032-DR) in 1μM PLX4032 (11).

### CCK-8

CCK-8 kit (Beyotime, C0037) was employed for examining the survival of PLX4032-DR. Specifically, the cells were inoculated into 96-well plates at 5*10^4^/well. After culturing for 24h, 48h, 72h, 96h, 10μL CCK-8 reagent was added to each well. After 1-h incubation, the absorbance was measured at 450nm with a microplate reader.

### Animal Model Construction and *In Vivo* UMMD Treatment

PLX4032-DR and Matrigel glue were mixed at a ratio of 1:1 and then subcutaneously injected into the left and right sides of 20 nude mice. When the tumor grew to a size large enough for further experiment, the mice were divided into 4 groups, namely, UMMD group, ultrasound group (US), micro Bubble group (MB) and control group (CON), with 5 mice in each group. According to a previous study (17), MBS was prepared, except for those in the US group and the CON group, each mouse was injected with MB suspension directly into the tumor, while the mice in the US group and the CON group were injected with the same amount of normal saline. Ultrasound treatment (1 mhz, 100% DC, 2.3W/cm^2^, 10s) in US group and UMMD group was performed on day 7, 8, 9, 11 and 13 after the MB perfusion. Tumor volume was measured and recorded once every 4 days after ultrasound treatment (V=0.5*longest axis*shortest axis^2^, mm). The mice were sacrificed 25 days after the perfusion, and the tumor volume and weight were carefully measured and calculated. The animal experiment was conducted strictly in accordance with the guidelines for the Care and Use of Laboratory Animals of the National Institutes of Health and was approved by the Ethics Committee of our hospital.

### RNA-Seq

Pooled sequencing of engrafted tumors from the CON and UMMD group were performed., and the library was constructed using the Illumina Truseq™ RNA sample prep Kit. The samples were sequenced on Illumina Hiseq 2000. Sequencing raw data have been uploaded to the Sequence Read Archive database (PRJNA706468).

### Ribo-Seq

Liquid nitrogen was used to freeze the mouse tumor tissues. By referring to a previous study (18), ribosomes were recovered after nuclease treatment, and we obtained the ribosome profiles on Ilumina NextSeq CN500% with a sequencing depth of 40M.

### Statistical Methods

Graphpad Prism 8.0 was applied for the analysis of experimental data. The count data were presented by percentage, and the chi-square test was used for the component comparison. Measurement data were expressed as mean ± standard deviation, t test was used for component comparison. Survival curve was drawn using Kaplan-Meier method, and survival difference was analyzed by log-rank test. Spearman test was employed to analyze the correlation between YAP1 expression and tumor weight. A *p*<0.05 was regarded as a statistically significant difference.

## Results

### Clinicopathological Characteristics of CMM Patients Was Irrelevant to YAP1 mRNA Expression

The YAP1 mRNA expression level in the tumor tissues of 64 CMM patients was determined to be 1.264 ± 0.105. According to the median expression level, the 64 patients were accordingly divided into high and low YAP1 mRNA expression groups. By comparing the relationship between mRNA expression of YAP1 and clinical pathological characteristics of the patients, we observed that YAP1 mRNA expression was irrelevant to the age, gender, lesion site, or cell subtype, etc. of CMM patients (*p*>0.05) (**Table 3**).

**Table 3:** Relationship between YAP1 mRNA and clinicopathological characteristics of CMM patients.

	Numbers	high expression YAP1 mRNA group (n = 32)	low expression YAP1 mRNA (n = 32)	χ^2^	*p*
Age				1.036	0.309
≤50	26	15(46.88)	11(34.38)		
>50	38	17(53.13)	21(65.63)		
Sex				3.090	0.079
Male	29	16(56.25)	13(34.38)		
Female	35	24(43.75)	11(65.63)		
Lesion				4.991	0.083
Neck	9	2(6.24)	7(21.88)		
Trunk	17	7(21.88)	10(31.24)		
Others	38	23(71.88)	15(46.88)		
Cell subtype				0.254	0.614
Epithelioid	36	19(59.38)	17(53.13)		
Fusocellular	28	13(40.62)	15(46.87)		
Organization Type				1.333	0.248
Nodular	16	10(31.25)	6(18.75)		
Others	48	22(68.75)	26(81.25)		
Clark classification				1.237	0.266
I-III	18	7(21.88)	11(34.38)		
IV-V	46	25(78.12)	21(65.62)		
Breslow Thickness (mm)				1.871	0.171
≤2	19	12(37.50)	7(21.88)		
>2	45	20(62.50)	25(78.12)		
BRAF (V600E) mutation				0.563	0.453
No	33	18(56.25)	15(46.88)		
Yes	31	14(43.75)	17(53.12)		
Tumor growth phase				1.896	0.169
Radial	10	7(21.88)	3(9.38)		
Vertical	54	25(78.12)	29(90.62)		
Mitosis count				0.277	0.599
≤5/HPF	42	22(68.75)	20(62.50)		
>5/HPF	22	10(31.25)	12(37.50)		

### Survival of CMM Patients Was Irrelevant to YAP1 mRNA Expression

**Figure 1:**
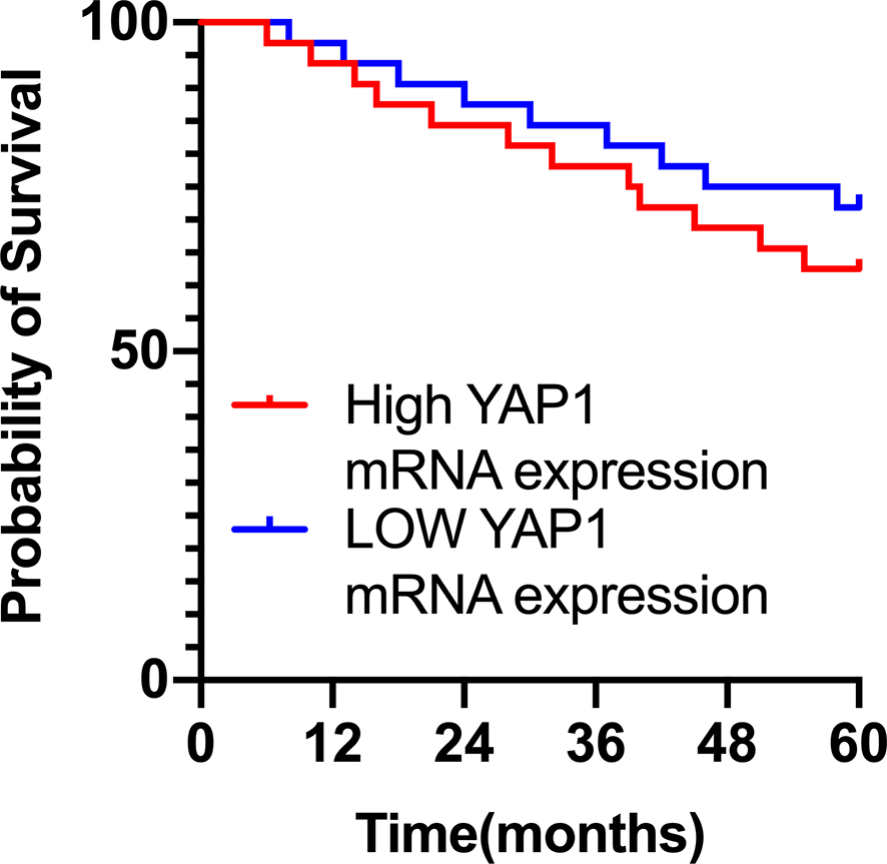
Survival curve of CMM patients in YAP1 mRNA high and low expression groups.

Further analysis of the relationship between the survival of CMM patients and YAP1 mRNA expression showed that although the 5-year survival rate of patients with high mRNA expression of YAP1 was slightly lower than that of those with low YAP1 mRNA expression, there was no statistical difference (Hazard Ratio (HR)=1.422, 95%) CI of ratio: 0.6043 to 3.347, *p*=0.422), indicating that the survival of CMM patients is irrelevant to the level of YAP1 mRNA expression (**Figure 1**).

### UMMD Treatment *In Vivo* Inhibited the Growth of Drug-Resistant CMM Tumors

**Figure 2:**
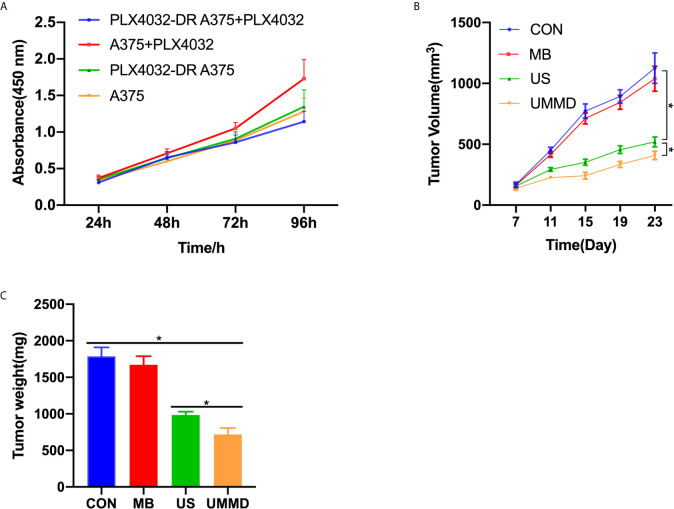
*In vivo* UMMD treatment inhibited the growth of drug-resistant CMM tumors. **(A)** CCK-8 assay was used to test the construction of drug-resistant CMM cell lines; **(B)** Changes in tumor volume; **(C)** The mice were sacrificed after 25 days of perfusion and weighed. *p < 0.05.

CCK-8 method was applied to detect the tumor cell growth, and the results showed that 1μM PLX4032 did not affect the cell viability of PLX4032-DR A375 (*p*>0.05), indicating a successful establishment of the drug-resistant CMM cell line, which was then adequately mixed with Matrigel and injected into the skin of the nude mice. Subsequently, the corresponding treatment was carried out. The data revealed that compared with the CON group, the MB group showed limited effect on the volume and weight of tumors (*p*>0.05). However, the tumor volume and weight of the US and UMMB groups were significantly lower than those of the MB and CON groups (*p*<0.05), noticeably, the tumor growth of the mice in the UMMB group was significantly inhibited (*p*<0.05) (**Figure 2**).

### UMMD Treatment *In Vivo* Promoted the Translation Efficiency of YAP1

**Figure 3:**
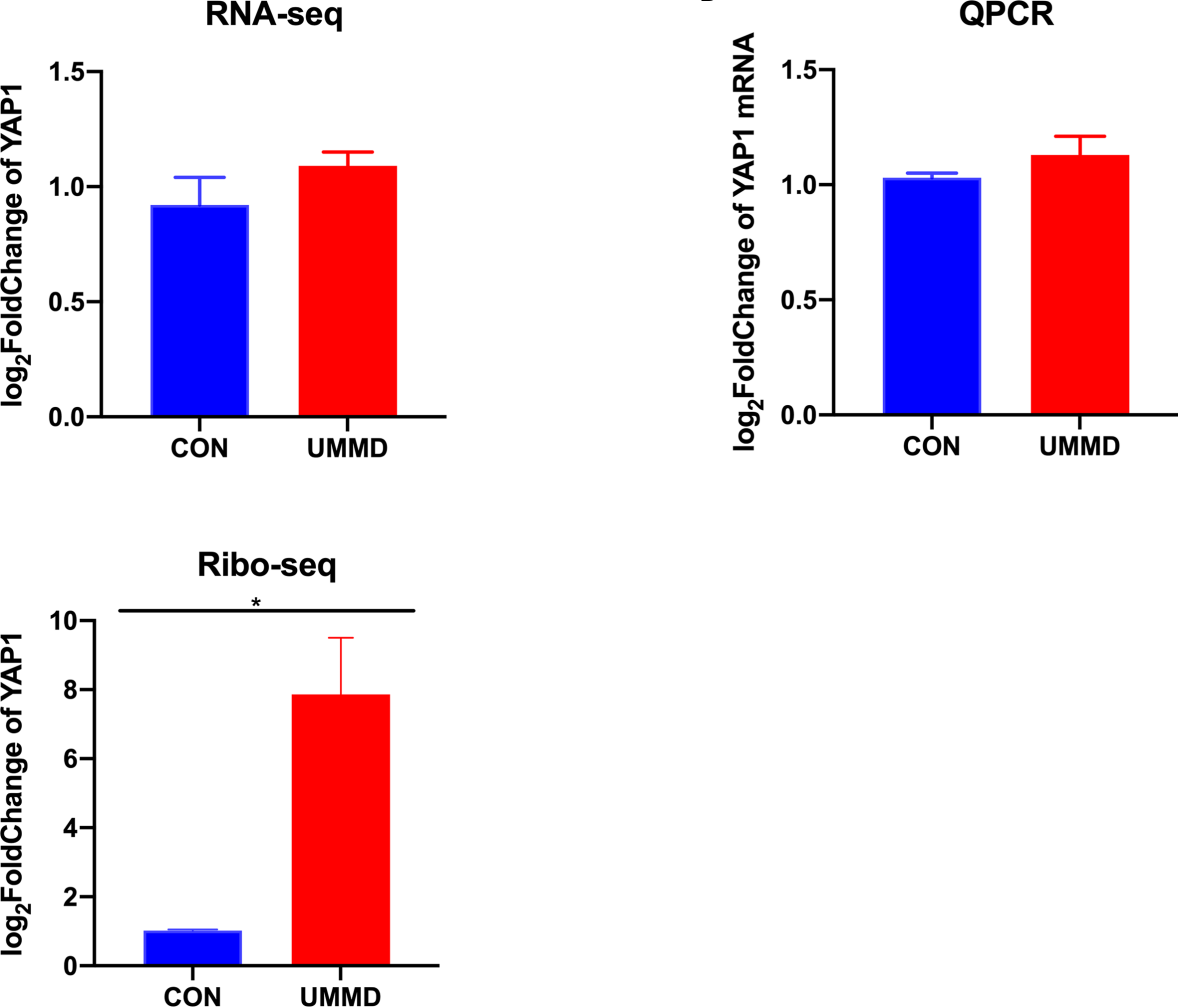
UMMD treatment *in vivo* promotes YAP1 translation efficiency. **(A)** RNA-seq detection of YAP1 expression in CMM mouse tumor tissues; **(B)** RT-qPCR detection of YAP1 mRNA expression in CMM mouse tumor tissues; **(C)** Ribo-seq detection of YAP1 expression in CMM mouse tumor tissues. *p < 0.05.

**Figure 4:**
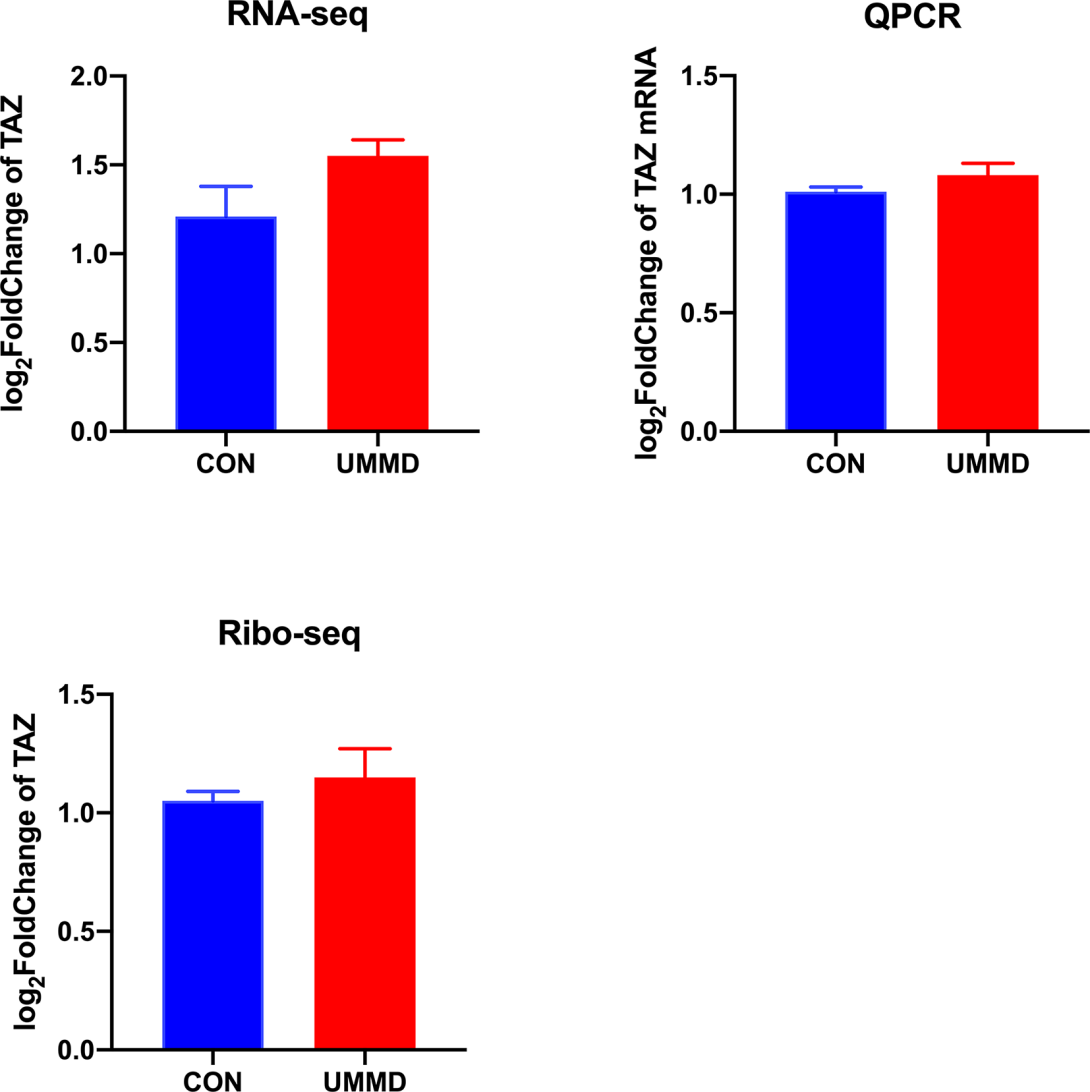
UMMD treatment *in vivo* does not affect TAZ expression. **(A)** RNA-seq detection of TAZ expression in CMM mouse tumor tissues; **(B)** RT-qPCR detection of TAZ mRNA expression in CMM mouse tumor tissues; **(C)** Ribo-seq detection of TAZ expression in CMM mouse tumor tissues.

RNA-seq was performed on the tumor tissue samples from all the CMM mice after corresponding treatment for determining the expression of YAP1, and it was found that the expression of YAP1 in the tumor tissues of each group of CMM mice did not change significantly (*p*>0.05), moreover, the results of RT-qPCR were consistent with those of RNA-seq (*p*>0.05). Ribo-seq was performed subsequently to further examine the effect of UMMD on YAP1 activity. Surprisingly, the results of Ribo-seq demonstrated that UMMD treatment can significantly up-regulate the expression of YAP1, especially UMMD, which has been found to have the most significant effect *(p*<0.05) (**Figure 3**). Spearman test results indicated that YAP1 expression in Ribo-seq was negatively correlated with the weight of CMM mice after treatment (r = -0.735, *p*<0.05), while YAP1 expression in RNA-seq and RT-qPCR was irrelevant to tumor tissue weight (r=-0.347, *p*>0.05; r = -0.299, *p*>0.05). However, as another important gene member on the Hippo signaling pathway, TAZ, we did not find obvious changes in its expression by UMMD treatment in the results of Ribo-seq(P > 0.05), as did RT qPCR with RNA SEQ (P > 0.05) (**Figure 4**).

## Discussion

Ultrasound and contrast agent technologies have made significant progress. Ultrasound technologies such as tumor ultrasound imaging and UMMD witnessed an increasing application in disease monitoring, diagnosis and treatment (19). US promotes pressure and temperature through cavitation, sonoporation, or thermal effects, which further enhances self-generated or exogenously introduced MB to rupture after expansion in the target area, resulting in changes in the permeability of tissues and cells (19, 20). In the past, UMMD was often used to mechanically dissolve thrombus substances in cardiovascular diseases such as ischemia/reperfusion injury, myocardial infarction, and hypertension, in addition, as a carrier of drugs and nucleic acids, it is expected to become an adjuvant therapy for the treatment of patients with heart disease (21, 22). Latest advances showed that UMMD, which focuses on the target area with high precision and non-invasiveness, can significantly reduce the dose and toxicity of drugs, improve the efficiency of drug and gene delivery, showing a high effectiveness in treating specific cancers such as hepatocellular carcinoma (23, 24). Study found that the implantation of MB into both the tumor tissues of melanoma cell line B16 and *in vivo* animal model followed by ultrasound treatment can hinder tumor growth and improve the survival rate of mice (17), and such findings are in line with the results of our current study. Consistently, we also found that UMMD treatment can inhibit CMM tumor growth.

The transcription of YAP1 and TAZ in the Hippo signaling pathway plays a critical role in mediating the resistance of major cancer treatment drugs, and is considered to play a major driving role in the development of resistance of BRAF- and KRAS- mutant cancer cells (12, 25, 26). Studies revealed that in highly invasive CMM cell lines, although TAZ expression is higher than YAP1 expression, both YAP1 and TAZ knockout can reduce the invasion and metastasis ability of CMM cells (27). However, in this study, the mRNA expression of YAP1 in tumor tissues was irrelevant to the clinicopathological characteristics or the survival of CMM patients. To explain such results, we speculated that on one hand, mRNA expression YAP1 cannot accurately reflect the transcriptional activity of YAP1, on the other hand, the specific role of YAP1 in CMM still remains unclear. Kim et al. (28) analyzed the clinical data of 88 local patients with uveal melanoma on The Cancer Genome Atlas (TCGA), and discovered that YAP1 activity is irrelevant to tumor size, tumor stage, gene mutation or other clinicopathological characteristics. The YAP1 nuclear-positive patients did not show a lower survival rate, which also supports our speculation that YAP1 activity was not a carcinogene as strong as described in other studies. A recent study also found (29) that targeted therapies of YAP1 and TAZ show anti-cancer effects on untreated human CMM cell lines, but such an effect was not observed in all patient-derived ectopic implant experiments.

Moreover, the activity of YAP1 in the epidermis may be independent of the Hippo signaling pathway, and is mainly controlled by adhesion junctions and downstream signal transduction of integrins as well as by the mechanical force transmitted and applied by the associated actin cytoskeleton. The mechanical signal, which affects the activity of YAP1 in the epidermis, plays a major role in the regulation of YAP/TAZ in fibroblasts (30). To further determine the expression of YAP1 in drug-resistant CMM and the effect of mechanical signals of UMMD on its expression, this research further applied RNA-seq, Ribo-seq and QPCR to explore the effects of UMMD treatment on CMM mouse tumor tissues *in vivo*. The results showed that only Ribo-seq UMMD and US showed the up-regulated YAP1 expression, with the expression of YAP1 in Ribo-seq inversely proportional to tumor growth, noticeably, the effect of UMMD treatment on YAP1 expression was the most obvious. Ribo-seq greatly facilitates the acquirement of ribosome distribution by sequencing “ribosome protected fragments”, and can analyze the translation efficiency and translation mode of genes on ribosomes (31). Ribo-seq serves as an indicator of instantaneous protein synthesis efficiency and stable transcription level. The core of this method is that translated ribosome protects a short fragment of mRNA from nuclease activation, thereby accurately recording the position in which translation takes place. Thus, Ribo-seq could sensitively and effectively detect protein changes in cells (32). The results of this experiment indicated that UMMD could affect the expression of YAP1 at a translation level.

Cavitation is a common mechanisms resulting in MB rupture in UMMD (19). At present, studies have found that the cavitation effect of UMMD can significantly reduce the survival of melanoma cells and improve the therapeutic effect of tumors (33). During the process of cavitation, when the oscillating MB gathers on the surface of cells or tissues, shear stress will be generated, leading to the deformation of MB or even rupture, and enhancing the temporary permeability of the cell membrane (34). When cells receive mechanical stress such as shear stress, they can regulate F-actin and AMOT in the cell to promote YAP1 dephosphorylation, successfully entering the nucleus and activating transcription (35, 36). On the other hand, it has been demonstrated that in EMT or metastatic cancer cells, the activation of YAP1 will up-regulate a variety of irons including acyl-CoA synthase long-chain family member 4 (ACSL 4) and transferrin receptor. Death regulators increase the sensitivity of cells to iron death (37). Affected by cell density, YAP1 could act as a new determinant of iron ptosis, while by promoting cell resistance to apoptosis, YAP1 will greatly increase the sensitivity of cancer cells to iron death and may be resistant to YAP1 activated drug-resistant metastatic tumors, thus showing a therapeutic potential in cancers (38, 39).

In summary, our research showed that the expression of YAP1 may not significantly promote the growth of CMM tumors. However, UMMD can greatly inhibit the growth of CMM tumors, and such an effect seems to be highly related to the expression of YAP1 in Ribo-seq. UMMD could promote YAP1 to enter the nucleus and increase the sensitivity of cells to iron death, thereby exerting a therapeutic effect, but the specific mechanism still requires further investigation in depth.

## Data Availability Statement

The datasets presented in this study can be found in online repositories. The names of the repository/repositories and accession number(s) can be found in the article/supplementary material.

## Ethics Statement

The studies involving human participants were reviewed and approved by Medical Ethics Committee of our hospital. The patients/participants provided their written informed consent to participate in this study. The animal study was reviewed and approved by Ethics Committee of Xiangya Hospital, Central South University.

## Author Contributions

All authors contributed to the article and approved the submitted version.

## Conflict of Interest

The authors declare that the research was conducted in the absence of any commercial or financial relationships that could be construed as a potential conflict of interest.

## References

[1] Siegel RL Miller KD Jemal A. Cancer statistics, 2019. CA: Cancer J Clin (2019) 69(1):7–34. 10.3322/caac.21551 30620402

[2] Hsueh EC DeBloom JR Lee J Chowdhury SM Sussman JJ Covington KR Middlebrook B. Interim analysis of survival in a prospective, multi-center registry cohort of cutaneous melanoma tested with a prognostic 31-gene expression profile test. J Hematol Oncol (2017) 10(1):152. 10.1186/s13045-017-0520-1 28851416 PMC5576286

[3] Saldanha G Yarrow J Elsheikh S O'Riordan M Uraiby H Bamford M. Development and initial validation of calculated tumor area as a prognostic tool in cutaneous malignant melanoma. JAMA Dermatol (2019) 155(8):890–8. 10.1001/jamadermatol.2019.0621 PMC6596334 31241720

[4] Kim DW Haydu LE Joon AY Bassett RL Jr. Siroy AE Tetzlaff MT. Clinicopathological features and clinical outcomes associated with TP53 and BRAFNon-V600 mutations in cutaneous melanoma patients. Cancer (2017) 123(8):1372–81. 10.1002/cncr.30463 PMC5384865 27911979

[5] Li Y Umbach DM Li L. Putative genomic characteristics of BRAF V600K versus V600E cutaneous melanoma. Melanoma Res (2017) 27(6):527–35. 10.1097/CMR.0000000000000388 PMC5669042 28858076

[6] Feller JK Yang S Mahalingam M. Immunohistochemistry with a mutation-specific monoclonal antibody as a screening tool for the BRAFV600E mutational status in primary cutaneous malignant melanoma. Modern Pathol (2013) 26(3):414–20. 10.1038/modpathol.2012.168 23041829

[7] Sosman JA Kim KB Schuchter L Gonzalez R Pavlick AC Weber JS. Survival in BRAF V600–mutant advanced melanoma treated with vemurafenib. New Engl J Med (2012) 366(8):707–14. 10.1056/NEJMoa1112302 PMC3724515 22356324

[8] Torres-Collado AX Knott J Jazirehi AR. Reversal of resistance in targeted therapy of metastatic melanoma: lessons learned from vemurafenib (BRAFV600E-Specific Inhibitor). Cancers (2018) 10(6):157. 10.3390/cancers10060157 PMC6025215 29795041

[9] Grijalva JL Huizenga M Mueller K Rodriguez S Brazzo J Camargo F. Dynamic alterations in Hippo signaling pathway and YAP activation during liver regeneration. Am J Physiol-Gastrointestinal Liver Physiol (2014) 307(2):G196–204. 10.1152/ajpgi.00077.2014 24875096

[10] Kim MH Kim J Hong H Lee S-H Lee J-K Jung E. Actin remodeling confers BRAF inhibitor resistance to melanoma cells through YAP/TAZ activation. EMBO J (2016) 35(5):462–78. 10.15252/embj.201592081 PMC4772854 26668268

[11] Fisher ML Grun D Adhikary G Xu W Eckert RL. Inhibition of YAP function overcomes BRAF inhibitor resistance in melanoma cancer stem cells. Oncotarget (2017) 8(66):110257–72. 10.18632/oncotarget.22628 PMC5746380 29299145

[12] Lin L Sabnis AJ Chan E Olivas V Cade L Pazarentzos E. The Hippo effector YAP promotes resistance to RAF-and MEK-targeted cancer therapies. Nat Genet (2015) 47(3):250–6. 10.1038/ng.3218 PMC4930244 25665005

[13] Wischhusen J Padilla F. Ultrasound-targeted microbubble destruction (UTMD) for localized drug delivery into tumor tissueJ. IRBM (2019) 40(1):10–5. 10.1016/j.irbm.2018.11.005

[14] Gao F Wu J Niu S Sun T Li F Bai Y. Biodegradable, pH-Sensitive Hollow Mesoporous Organosilica Nanoparticle (HMON) with Controlled Release of Pirfenidone and Ultrasound-Target-Microbubble-Destruction (UTMD) for Pancreatic Cancer Treatment. Theranostics (2019) 9(20):6002–18. 10.7150/thno.36135v PMC6735371 31534533

[15] Sun M Spill F Zaman MH. A computational model of YAP/TAZ mechanosensingJ. Biophys J (2016) 110(11):2540–50. 10.1016/j.bpj.2016.04.040 PMC4922562 27276271

[16] Li W Wang W Uren PJ Penalva LOF Smith AD. Riborex: fast and flexible identification of differential translation from Ribo-seq data. Bioinformatics (2017) 33(11):1735–7. 10.1093/bioinformatics/btx047 PMC5860393 28158331

[17] Jang KW Seol D Ding L Lim TH Frank JA Martin JA. Ultrasound-Mediated Microbubble Destruction Suppresses Melanoma Tumor Growth. Ultrasound Med Biol (2018) 44(4):831–9. 10.1016/j.ultrasmedbio2017.12.011 PMC5826859 29361373

[18] Kiss DL Baez W Huebner K Bundschuh R Schoenberg DR. Impact of FHIT loss on the translation of cancer-associated mRNAs. Mol Cancer (2017) 16(1):179. 10.1186/s12943-017-0749-x 29282095 PMC5745650

[19] Izadifar Z Babyn P Chapman D. Ultrasound cavitation/microbubble detection and medical applicationsJ. J Med Biol Engineer (2019) 39(3):259–76. 10.1007/s40846-018-0391-0

[20] Chowdhury SM Abou-Elkacem L Lee T Dahl J Lutz AM. Ultrasound and microbubble mediated therapeutic delivery: Underlying mechanisms and future outlook. J Controlled Release (2020) 326:75–90. 10.1016/j.jconrel.2020.06.008 32554041

[21] Qian L Thapa B Hong J Zhang Y Zhu M Chu M. The present and future role of ultrasound targeted microbubble destruction in preclinical studies of cardiac gene therapy. J Thorac Dis (2018) 10(2):1099–111. 10.21037/jtd2018.01.101 PMC5864676 29607187

[22] Rix A Curaj A Liehn E Kiessling F. Ultrasound microbubbles for diagnosis and treatment of cardiovascular diseasesC. Semin Thromb Hemost (2020) 46(05):545–52. 10.1055/s-0039-1688492 31096311

[23] Chowdhury SM Wang TY Bachawal S Devulapally R Choe JW Elkacem LA. Ultrasound-guided therapeutic modulation of hepatocellular carcinoma using complementary microRNAs. J Controlled Release (2016) 238:272–80. 10.1016/j.jconrel.2016.08.005 PMC5185600 27503707

[24] Mullick Chowdhury S Lee T Willmann JK. Ultrasound-guided drug delivery in cancer. Ultrasonography (2017) 36(3):171–84. 10.14366/usg.17021 PMC5494871 28607323

[25] Nguyen CDK Yi C. YAP/TAZ Signaling and Resistance to Cancer Therapy. Trends Cancer (2019) 5(5):283–96. 10.1016/j.trecan.2019.02.010 PMC6557283 31174841

[26] Shao DD Xue W Krall EB Bhutkar A Piccioni F Wang X. KRAS and YAP1 converge to regulate EMT and tumor survival. Cell (2014) 158(1):171–84. 10.1016/j.cell.2014.06.004 PMC4110062 24954536

[27] Nallet-Staub F Marsaud V Li L Gilbert C Dodier S Bataille V. Pro-invasive activity of the Hippo pathway effectors YAP and TAZ in cutaneous melanoma. J Invest Dermatol (2014) 134(1):123–32. 10.1038/jid.2013.319 PMC3938155 23897276

[28] Kim YJ Lee SC Kim SE Kim SH Kim SK Lee CS. YAp Activity is not Associated with Survival of Uveal Melanoma patients and cell Lines. Sci Rep (2020) 10(1):1–9. 10.1038/s41598-020-63391-z 32277165 PMC7148330

[29] Zhang X Tang JZ Vergara IA Zhang Y Szeto P Yang L. Somatic hypermutation of the YAP oncogene in a human cutaneous melanoma. Mol Cancer Res (2019) 17(7):1435–49. 10.1158/1541-7786.MCR-18-0407 30833299

[30] Rognoni E Walko G. The roles of YAP/TAZ and the hippo pathway in healthy and diseased skin. Cells (2019) 8(5):411. 10.3390/cells8050411 PMC6562585 31058846

[31] Cui H Hu H Zeng J Chen T. DeepShape: estimating isoform-level ribosome abundance and distribution with Ribo-seq data. BMC Bioinf (2019) 20(24):1–13. 10.1186/s12859-019-3244-0 PMC6923924 31861979

[32] Brar GA Weissman JS. Ribosome profiling reveals the what, when, where and how of protein synthesis. Nat Rev Mol Cell Biol (2015) 16(11):651–64. 10.1038/nrm4069 PMC5522010 26465719

[33] Shanei A Akbari-Zadeh H Attaran N Salamat MR Baradaran-Ghahfarokhi M. Effect of targeted gold nanoparticles size on acoustic cavitation: An in vitro study on melanoma cells. Ultrasonics (2020) 102:106061. 10.1016/j.ultras.2019.106061 31948804

[34] Tu J Zhang H Yu J Liufu C Chen Z. Ultrasound-mediated microbubble destruction: a new method in cancer immunotherapy. Onco Targets Ther (2018) 11:5763–75. 10.2147/OTTS171019 PMC6140758 30254469

[35] Biggins JS Royer C Watanabe T Srinivas S. Towards understanding the roles of position and geometry on cell fate decisions during preimplantation developmentC. Semin Cell Dev Biol (2015) 47:74–9. 10.1016/j.semcdb.2015.09.006 PMC4683091 26349030

[36] Nakajima H Yamamoto K Agarwala S Terai K Fukui H Fukuhara S. Flow-dependent endothelial YAP regulation contributes to vessel maintenance. Dev Cell (2017) 40(6):523–536. e6. 10.1016/j.devcel.2017.02.019 28350986

[37] Wu J Minikes AM Gao M Bian H Li Y Stockwell BR. Intercellular interaction dictates cancer cell ferroptosis via NF2-YAP signalling published correction appears in Nature. Nature (2019) 572(7769):402–6. 10.1038/s41586-019-1426-6 PMC6697195 31341276

[38] Sun T Chi JT. Regulation of ferroptosis in cancer cells by YAP/TAZ and Hippo pathways: the therapeutic implications. Genes Dis (2020) 572(7770). 10.1016/j.gendis.2020.05.004 PMC8093643 33997171

[39] Yang WH Chi JT. Hippo pathway effectors YAP/TAZ as novel determinants of ferroptosis. Mol Cell Oncol (2020) 7(1):1699375. 10.1080/23723556.2019.1699375 31993503 PMC6961671

